# Supplementary material for: Text messaging reminders for influenza vaccine in primary care: protocol for a cluster randomised controlled trial (TXT4FLUJAB)
Source: BMJ Open. 2014 Apr 30;4(5):e004633. doi: 10.1136/bmjopen-2013-004633 (PMC4025454; doi:10.1136/bmjopen-2013-004633)
Supplement: Author's manuscript [file bmjopen-2013-004633.draft_revisions.pdf]

Text messaging reminders for influenza vaccine in primary care: protocol for a cluster randomised controlled trial (TXT4FLUJAB)

Journal:	*BMJ Open*
Manuscript ID:	bmjopen-2013-004633
Article Type:	Protocol
Date Submitted by the Author:	06-Dec-2013

Complete List of Authors:	Herrett, Emily; London School of Hygiene and Tropical Medicine, Non communicable disease epidemiology<br>van Staa, Tjeerd; Utrecht University,<br>Free, Caroline; London School of Hygiene and Tropical Medicine, Public Health interventions Unit<br>Smeeth, Liam; London School of Hygiene and Tropical Medicine, Epidemiology and Population Health

**Primary Subject Heading**:	Health services research
Secondary Subject Heading:	Infectious diseases
Keywords:	PRIMARY CARE, EPIDEMIOLOGY, PREVENTIVE MEDICINE

SCHOLARONE™ Manuscripts

For peer review only

Text messaging reminders for influenza vaccine in primary care: protocol for a cluster randomised controlled trial (TXT4FLUJAB)

Dr Emily Herrett (corresponding author, emily.herrett@lshtm.ac.uk)[1], Professor Tjeerd van Staa (tjeerd.vanstaa@lshtm.ac.uk)[1,2], Dr Caroline Free (caroline.free@lshtm.ac.uk)[1], Professor Liam Smeeth (liam.smeeth@lshtm.ac.uk)[1]

[1] London School of Hygiene and Tropical Medicine, Keppel Street, London, WC1E 7HT, UK

[2] Division of Pharmacoepidemiology and Clinical Pharmacology, Department of Pharmaceutical Sciences, Faculty of Sciences, Utrecht Institute for Pharmaceutical Sciences, Utrecht University, Utrecht, Netherlands

For peer review only

For peer review only - http://bmjopen.bmj.com/site/about/guidelines.xhtml

Abstract

Introduction: The UK government recommends that at least 75% of people aged under 65 with certain conditions receive an annual influenza vaccination. Primary care practices often fail short of this target and strategies to increase vaccine uptake are required. Text messaging reminders are already used in 30% of practices to remind patients about vaccination but there has been no trial addressing their effectiveness in increasing influenza vaccine uptake in the UK.

The aims of the study are (i) to implement a cluster randomised trial to test the effectiveness of using a text messaging influenza vaccine reminder in achieving an increase in influenza vaccine uptake in patients aged 18 to 65 with chronic condition compared to standard care; and (ii) to develop the methodology for conducting cluster randomised trials of text messaging interventions utilising routine electronic health records.

Methods and analysis: This cluster randomised trial will recruit general practices across three settings in English primary care (Clinical Practice Research Datalink, ResearchOne and London iPLATO text messaging software users) and randomise them to either standard care or a text messaging campaign to eligible patients. Flu vaccine uptake will be ascertained using routinely collected, anonymised electronic patient records. This protocol outlines the proposed study design and analysis methods.

Ethics and dissemination: Results of this study will determine the effectiveness of text messaging vaccine reminders in primary care in increasing influenza vaccine uptake, and will strengthen the methodology for using electronic health records in cluster randomised trials of text messaging interventions. This trial was approved by Surrey Borders Ethics Committee (13/LO/0872). The trial results will be disseminated at national conferences and published in a peer reviewed medical journal. The results will also be distributed to the Primary Care Research Network and to all participating general practices.

For peer review only

For peer review only - http://bmjopen.bmj.com/site/about/guidelines.xhtml

Trial registration: This study is registered at controlled-trials.com ISRCTN48840025

Strengths and limitations of this study

Strengths

- This trial is the first to evaluate the effectiveness of text messaging for influenza vaccine reminders in English primary care.
- Text messaging is cheap, quick, and has shown to be effective for appointment reminders.
- The study requires minimal input from practices as it utilises routinely collected electronic health records to gather vaccine uptake data.

Limitations

- There may be some contamination if general practices in the standard care arm choose to send a text message.

For peer review only

For peer review only - http://bmjopen.bmj.com/site/about/guidelines.xhtml

Introduction

Influenza morbidity and mortality causes a substantial financial burden to the NHS and to the UK as a whole. Influenza vaccine is safe and effective but is required annually because the circulating strain of virus changes each year. In the UK in 2012, the Chief Medical Officer (CMO) has recommended that at least 75% of elderly people (aged 65+) and 75% people under 65 with certain chronic conditions (e.g. chronic heart disease, diabetes, asthma, etc) should be vaccinated.

While primary care practices are achieving these targets for elderly patients (74% vaccinated in 2011/12), those set for patients with chronic conditions are not being met and have shown no substantial increases in the past decade[1] and vaccine uptake in 2011/12 was 51.6% across the risk groups, see Supplementary Table 1) Therefore strategies to increase flu vaccine uptake in these patients are required. Several randomised trials have demonstrated the effectiveness of flu vaccine reminders delivered to patients by letter, postcard or telephone.[2 3] However, use of text messaging in the NHS for appointment reminders is increasing as it is cheap, quick and effective. Text messaging is already used in roughly 30% of practices to remind patients about their flu vaccine[4] but there has been no trial addressing its effectiveness. Trials of text messaging in the US have shown some success as vaccine reminders, but whether there is an effect in UK primary care is unknown.

Therefore, we are performing a randomised trial of a text messaging flu vaccine reminder in patients aged under 65 who have a chronic condition. We will randomise whole primary care practices to either standard care or a text messaging campaign to eligible patients.

Aims and objectives

The aims of the study are (1) to implement a cluster randomised trial to test the effectiveness of using a text messaging influenza vaccine reminder in achieving an increase in influenza vaccine uptake in patients aged 18 to 65 with chronic condition compared to standard care;

For peer review only

and (2) to develop the methodology for conducting cluster randomised trials of text messaging interventions using routine electronic health records.

Specific objectives are: (i) to evaluate the effect of text messaging influenza vaccination reminders in patients under 65 with chronic conditions;(ii) to evaluate the feasibility of recruiting and randomising practices to a text messaging intervention; (iii) to evaluate the feasibility of practice delivery of a text message intervention to eligible patients within a primary care setting, and (iv) to evaluate the feasibility of ascertaining practice data regarding text message delivery and flu vaccine uptake.

For peer review only

Methods

Study design

A cluster randomised trial[5 6] of a text messaging influenza vaccination reminder in primary care.

Study population

This study is taking place in English primary care, with general practices recruited from three settings: (i) the Clinical Practice Research Datalink (CPRD),[7] a primary care database based on Vision software and covering 8% of the UK population; (ii) ResearchOne, a primary care database based on TPP SystmOne software[8] and covering 7.6% of the UK population; (iii) iPLATO text messaging software users in London.[9]

Inclusion criteria

Eligible practices must currently use a text messaging system to communicate with patients about issues other than influenza vaccination (CPRD and London practices must use iPLATO[9], ResearchOne practices must use TPP SystmOne text messaging software. Practices using other text messaging software will be excluded). Practices that used text messages for influenza vaccination reminders in the 2012/13 influenza season will be excluded.

Recruitment

Eligible practices in the CPRD (n=40) will be identified using the electronic health record and invited to the trial. All TPP SystmOne practices (n=2073) and London-based iPLATO practices (n=460) will be invited to the trial by the Primary Care Research Network. London iPLATO practices will also be contacted by iPLATO and TPP SystmOne practices will be targeted through SystmOne communications.

For peer review only - http://bmjopen.bmj.com/site/about/guidelines.xhtml
For peer review only

Sample size calculation

The sample size calculation is based on a comparison between influenza vaccine uptake in the intervention group, in which a text message influenza vaccination reminder will be sent to eligible patients, and influenza vaccine uptake in the standard care group. A cluster-level analysis of the practice-specific proportions will be undertaken and the sample size calculation estimates the number of practices required for the study. A systematic review of reminders for influenza vaccination reported that reminders (of any type) for influenza vaccine among adults increased the uptake, with an odds ratio of 1.66, 95% confidence interval 1.31-2.09). However, a text messaging vaccine reminder trial among children in the USA found only a 9% increase (RR=1.09, 95% confidence interval 1.04-1.15).[10] Therefore we have chosen to power our study for risk ratio of 1.075, representing an increase from 54% (uptake in the CPRD 2012/13) to 58%.

At 90% power and 5% significance, with an ICC by general practice of 0.024 and an average of 750 eligible patients per practice, we will require 100 practices (50 per arm) to identify a 7.5% increase in vaccine uptake from 54% to 58%. To account for differences in the number of eligible patients per practice and in the proportion with mobile phone numbers recorded, we have chosen to recruit and randomise 150 practices to the study.

Assignment of interventions

Practices will be randomised on a 1:1 basis to the intervention and standard care groups. We will use block randomisation within each setting. In the CPRD and ResearchOne, we will stratify by region.

Box 1
"Hello *PATIENT NAME*, to reduce your risk of serious health problems from flu we recommend vaccination. Call *PRACTICE PHONE NUMBER* to book. *PRACTICE NAME"*

For peer review only - http://bmjopen.bmj.com/site/about/guidelines.xhtml

Blinding

Practices in this study will be randomised to intervention or standard care groups, but general practice staff will not be blinded. However, academic investigators and trial statisticians will manage and review data without knowledge of the allocation.

Interventions

Practices will be allocated to an intervention or standard care arm. Those in the intervention arm will be asked to send an influenza vaccination text message reminder to their under 65 at-risk patients. This includes patients who are under 65 with chronic heart disease, chronic neurological disease, diabetes, chronic kidney disease, chronic liver disease, chronic respiratory disease, immunosuppression, as set out by the Chief Medical Officer.[11] Practices will identify eligible patients based on their electronic medical records using their established systems. The message content that practices will be asked to send is shown in Box 1.

Practices will receive guidance notes regarding delivery of the message (content, timing, eligible patients) and an incentive payment of £200. An online questionnaire will be sent to practices in the intervention arm to ascertain feedback from practice staff about delivery of the intervention.

Practices allocated to the standard care group will be asked to continue with their seasonal flu campaign as planned.

Sub-study for patient feedback

A sub-study will be conducted in two participating intervention practices. Each practice will send an anonymous questionnaire to patients that were targeted with the text message reminder. This questionnaire will address patient acceptability of the text message.

For peer review only - http://bmjopen.bmj.com/site/about/guidelines.xhtml

Outcomes

The aims of the study are (1) to implement a cluster randomised trial to test the effectiveness of using a text messaging influenza vaccine reminder in achieving an increase in influenza vaccine uptake in patients aged 18 to 65 with chronic condition compared to standard care; and (2) to develop the methodology for conducting cluster randomised trials of text messaging interventions using routine electronic health records.

To address aim 1, we will examine the proportion of eligible patients that were sent a text message to remind them about flu vaccine, and the proportion that received the influenza vaccination. This will be stratified by risk group, age and sex.

To address aim 2, we will measure:

- practice recruitment rate;
- the proportion of intervention practices that sent a text message to eligible patients;
- the proportion of eligible patients that were sent a message;
- the proportion of practices reporting any problems with delivering the message,
- the availability of data to examine text message receipt and vaccine uptake in each risk group,
- the time and cost required to gather data,

In the patient sub-study, we will also examine the acceptability of text messaging to patients, by measuring the proportion of patients that reported problems with the text message.

Data collection methods

All patient-level outcomes will be evaluated through analysis of routinely collected data. In practices that contribute data to the CPRD or ResearchOne, relevant data will be extracted from the database using specified Read codes. Within London practices using iPLATO software, data will be extracted by iPLATO using secure servers.

For peer review only - http://bmjopen.bmj.com/site/about/guidelines.xhtml

For peer review only

We will extract data on age, sex, clinical risk group (based on historical data from the medical record and prescription data), vaccination reminder type (text, letter, phone call, face to face), vaccination uptake, death, transfer out of practice, flu and flu-like illness, and hospital admissions. Uptake and patient outcomes will be analysed from 1st September to 31st December 2013. All of these data items are routinely collected by general practices and stored in their electronic health record, and follow-up for practices will be complete using this method. Time and cost of data collection using these methods will be estimated.

We will record the number of practices approached, recruited and analysed. An online questionnaire, emailed to each practice in the intervention group, will ascertain practice acceptability of the intervention. The postal questionnaire to patients in the sub-study will estimate patient acceptability.

Statistical methods

A cluster-level analysis will be performed using practice specific proportions as observations. We will compare vaccine uptake in the intervention and standard care groups using a t test, with the size of clusters taken into account. A series of descriptive statistics will describe the methodological outcomes.

Sensitivity and subgroup analyses

Our primary analysis will be an intention to treat analysis. However, to account for any contamination between the standard care and intervention arms, we will carry out a per-protocol analysis.

Where available, we will also measure the difference in vaccine uptake, comparing practices that used the exact wording of the message in the study protocol with practices that used an alternative message. We hypothesise that practices using our message (based on behavioural theory) will have a higher uptake than an alternative designed by the practice. As this will be a non-randomised comparison, we will adjust for confounders.

For peer review only - http://bmjopen.bmj.com/site/about/guidelines.xhtml

Finally, we will compare the effectiveness of the text message based on the time of day that it was sent to patients. We hypothesise that messages sent in the late afternoon will have more effect that those sent at earlier times of day, when patients may not have the time to respond.

Hawthorne effect

The study design allows an evaluation of the generalisibility of the study population; practices that take part in the study can be compared to other practices outside of the trial that contribute data to ResearchOne and CPRD. We will test whether participation in the trial changes the behaviour of practices in their use of text messaging (Hawthorne effect).

This study will provide evidence regarding the effectiveness of text messaging reminders for influenza vaccine in patients under 65 with chronic conditions. The methodology here will be applied to future cluster randomised trials of text messaging interventions within electronic heath records.

Ethics and dissemination

Ethical issues

Approvals

The study protocol has received a favourable opinion from the National Research Ethics Service Committee – Surrey Borders (REC number 13/LO/0872) and has received assurance from participating Primary Care Trusts/ Clinical Commissioning Groups.

Informed consent

This is a cluster trial and informed consent will be obtained from GPs, who will act as the guardian of the cluster. A signed and dated informed consent form is required for participation in the trial. The risks and benefits of participation will be explained and GPs are free to decline to participate in the trial. Individual consent will not be sought. This is justified

For peer review only - http://bmjopen.bmj.com/site/about/guidelines.xhtml

For peer review only

because participating practices already use text messaging to communicate with patients, though not systematically for influenza. Patients within these practices will have the opportunity to opt out of receiving messages if they do not wish to be contacted in this way.

Patient confidentiality

All patient-level data accessed in this study will be stripped of personal identifiers.

Burden and risk to practices

Text messaging is widely used in everyday primary care and the only substantive change from non-study is the random allocation of a defined text message regarding influenza vaccine rather than the practice's own seasonal flu campaign. Patients can opt-out of receiving text messages from their practices, and as part of this study, we will monitor patient and practice complaints regarding the text message. Patients registered at practices in the intervention arm will receive one text message regarding their flu vaccine to minimise irritation to patients.

Participating practices must spend time identifying eligible patients and sending a text message. We are reducing this burden by (i) asking practices to use established procedures and software to identify eligible patients for vaccination. This is a task that they would perform annually in non-study conditions; (ii), we are including only practices that are familiar with the text messaging software, which minimises time spent in familiarisation with the software and sending messages; (iii) additional support is available to any practice through a guidance document and/or dedicated support helpline; (iv) we are providing an incentive payment (£200) to practices in the intervention group to encourage participation.

Practices may experience increased demand for influenza vaccination appointments or run out of vaccine supplies sooner than expected. The increase in uptake is unlikely to be large and will help the practice to achieve their Quality and Outcomes Framework target for vaccination, for which they receive payment.

For peer review only - http://bmjopen.bmj.com/site/about/guidelines.xhtml

Benefits for practices

Practices in the intervention arm will receive a £200 incentive to participate. If the text message is effective, then increased uptake will result in additional incentive payment to GPs by the government and will help them reach their Quality and Outcomes Framework targets. The results of this trial will allow practices to make an informed decision about spending of their budget on the seasonal influenza campaign.

Dissemination plans

The results of this trial will be published according to the guidelines of the CONSORT statement. The results will be published in a peer reviewed medical journal and presented at national conferences. The results will also be distributed to the Primary Care Research Network and to all participating general practices.

For peer review only

For peer review only - http://bmjopen.bmj.com/site/about/guidelines.xhtml

References

1. Begum F, Pebody R, Department of Health. Seasonal influenza vaccine uptake amongst GP patient groups in England: Winter season 2011/12, 2012.
2. Jacobson Vann Julie C, Szilagyi P. Patient reminder and recall systems to improve immunization rates. Cochrane Database of Systematic Reviews 2005; (3). http://onlinelibrary.wiley.com/doi/10.1002/14651858.CD003941.pub2/abstract.
3. Szilagyi PG, Bordley C, Vann JC, et al. Effect of patient reminder/recall interventions on immunization rates: A review. JAMA 2000;**284**(14):1820-7
4. Dexter LJ, Teare MD, Dexter M, Siriwardena AN, Read RC. Strategies to increase influenza vaccination rates: outcomes of a nationwide cross-sectional survey of UK general practice. BMJ Open 2012;**2**(3) doi: 10.1136/bmjopen-2011-000851[published Online First: Epub Date]|.
5. Campbell MK, Piaggio G, Elbourne DR, Altman DG. Consort 2010 statement: extension to cluster randomised trials. BMJ 2012;**345**:e5661 doi: 10.1136/bmj.e5661[published Online First: Epub Date]|.
6. Weijer C, Grimshaw JM, Eccles MP, et al. The Ottawa Statement on the Ethical Design and Conduct of Cluster Randomized Trials. PLoS Med 2012;**9**(11):e1001346 doi: 10.1371/journal.pmed.1001346[published Online First: Epub Date]|.
7. The Clinical Practice Research Datalink (CPRD) (www.cprd.com) accessed 1 December 2013.
8. TPP SystmOne (www.tpp-uk.com) accessed 1 December 2013.
9. iPLATO (www.iplato.net) accessed 17 April 2012.
10. Stockwell MS, Kharbanda EO, Martinez RA, Vargas CY, Vawdrey DK, Camargo S. Effect of a text messaging intervention on influenza vaccination in an urban, low-income pediatric and adolescent population: a randomized controlled trial. JAMA 2012;**307**(16):1702-8
11. McBride M, McArdle C, Timoney M. From the Chief Medical Officer: The seasonal influenza vaccination programme 2013/14. Department of Health, Social Services and Public Safety., 2013.

For peer review only

For peer review only - http://bmjopen.bmj.com/site/about/guidelines.xhtml

Authors' contributions

All authors were involved in the study design and in writing the manuscript.

Funding statement

This work was supported by the Wellcome Trust grant number 098504/Z/12/Z.

Competing interests statement

The authors declare no competing interests.

Acknowledgements

We would like to thank the LSHTM Clinical Trials Unit, Tim Collier, Noclor, Public Health England, the Cabinet Office, the Primary Care Research Network, the Department of Health, the Clinical Practice Research Datalink, ReserachOne, and iPLATO for their guidance and support.

For peer review only

Supplementary Table 1

**Risk groups for influenza vaccination**
Chronic respiratory disease
Chronic heart disease
Chronic renal disease
Diabetes
Immunosuppression
Liver disease
Chronic neurological disease
Carer
Pregnancy*

*not included in the TXT4FLUJAB study for ethical reasons

For peer review only

BMJ Open

Text messaging reminders for influenza vaccine in primary care: protocol for a cluster randomised controlled trial (TXT4FLUJAB)

Journal:	*BMJ Open*
Manuscript ID:	bmjopen-2013-004633.R1
Article Type:	Protocol
Date Submitted by the Author:	07-Apr-2014
Complete List of Authors:	Herrett, Emily; London School of Hygiene and Tropical Medicine, Non communicable disease epidemiology<br>van Staa, Tjeerd; Utrecht University,<br>Free, Caroline; London School of Hygiene and Tropical Medicine, Public Health interventions Unit<br>Smeeth, Liam; London School of Hygiene and Tropical Medicine, Epidemiology and Population Health
**Primary Subject Heading**:	Health services research
Secondary Subject Heading:	Infectious diseases
Keywords:	PRIMARY CARE, EPIDEMIOLOGY, PREVENTIVE MEDICINE

SCHOLARONE™
Manuscripts

For peer review only

Text messaging reminders for influenza vaccine in primary care: protocol for a cluster randomised controlled trial (TXT4FLUJAB)

For peer review only

Dr Emily Herrett (corresponding author, emily.herrett@lshtm.ac.uk)[1], Professor Tjeerd van Staa (tjeerd.vanstaa@lshtm.ac.uk)[1,2], Dr Caroline Free (caroline.free@lshtm.ac.uk)[1], Professor Liam Smeeth (liam.smeeth@lshtm.ac.uk)[1]

[1] London School of Hygiene and Tropical Medicine, Keppel Street, London, WC1E 7HT, UK

[2] Division of Pharmacoepidemiology and Clinical Pharmacology, Department of Pharmaceutical Sciences, Faculty of Sciences, Utrecht Institute for Pharmaceutical Sciences, Utrecht University, Utrecht, Netherlands

For peer review only - http://bmjopen.bmj.com/site/about/guidelines.xhtml

Abstract

Introduction: The UK government recommends that at least 75% of people aged under 65 with certain conditions receive an annual influenza vaccination. Primary care practices often fall short of this target and strategies to increase vaccine uptake are required. Text messaging reminders are already used in 30% of practices to remind patients about vaccination but there has been no trial addressing their effectiveness in increasing influenza vaccine uptake in the UK.

The aims of the study are (i) to develop the methodology for conducting cluster randomised trials of text messaging interventions utilising routine electronic health records; and (ii) to assess the effectiveness of using a text messaging influenza vaccine reminder in achieving an increase in influenza vaccine uptake in patients aged 18 to 65 with chronic conditions, compared to standard care.

Methods and analysis: This cluster randomised trial will recruit general practices across three settings in English primary care (Clinical Practice Research Datalink, ResearchOne and London iPLATO text messaging software users) and randomise them to either standard care or a text messaging campaign to eligible patients. Flu vaccine uptake will be ascertained using routinely collected, anonymised electronic patient records. This protocol outlines the proposed study design and analysis methods.

Ethics and dissemination: Results of this study will determine the effectiveness of text messaging vaccine reminders in primary care in increasing influenza vaccine uptake, and will strengthen the methodology for using electronic health records in cluster randomised trials of text messaging interventions. This trial was approved by Surrey Borders Ethics Committee (13/LO/0872). The trial results will be disseminated at national conferences and published in a peer reviewed medical journal. The results will also be distributed to the Primary Care Research Network and to all participating general practices.

For peer review only - http://bmjopen.bmj.com/site/about/guidelines.xhtml

Trial registration: This study is registered at controlled-trials.com ISRCTN48840025, July 2013.

Strengths and limitations of this study

Strengths

- This trial is the first to evaluate the effectiveness of text messaging for influenza vaccine reminders in English primary care.
- Text messaging is cheap, quick, and has shown to be effective for appointment reminders.
- The study requires minimal input from practices as it utilises routinely collected electronic health records to gather vaccine uptake data.

Limitations

- There may be some contamination if general practices in the standard care arm choose to send a text message.

For peer review only

For peer review only - http://bmjopen.bmj.com/site/about/guidelines.xhtml

Introduction

Influenza morbidity and mortality causes a substantial financial burden to the NHS and to the UK as a whole. Influenza vaccine is safe and effective but is required annually because the circulating strain of virus changes each year. In the UK in 2012, the Chief Medical Officer (CMO) recommended that at least 75% of elderly people (aged 65+) and 75% people under 65 with certain chronic conditions (e.g. chronic heart disease, diabetes, asthma, etc) should be vaccinated.[1]

While many primary care practices are achieving these targets for elderly patients (74% vaccinated in 2011/12), those set for patients with chronic conditions are not being met and have shown no substantial increases in the past decade;[2] vaccine uptake in 2011/12 was 51.6% across the risk groups (see Supplementary Table 1). Barriers to influenza vaccination in the UK include the lack of recommendation for vaccination by a healthcare worker,[3] lack of awareness of eligibility for vaccination,[4] low perceived susceptibility to and severity of influenza,[3-5] beliefs that the effectiveness of the vaccine is low, and beliefs about its side effects, safety, and pain.[3 5] Strategies to increase flu vaccine uptake are required.

Several randomised trials have demonstrated the effectiveness of flu vaccine reminders delivered to patients by letter, postcard or telephone.[6 7] However, use of text messaging in the NHS for appointment reminders is increasing as it is cheap, quick and effective. Text messaging is already used in roughly 30% of practices to remind patients about their flu vaccine[8] but there has been no trial addressing its effectiveness in this context. Trials of text messaging in the US have shown some success as vaccine reminders,[9] but whether there is an effect in UK primary care is unknown.

Therefore, we are performing a randomised trial of a text messaging flu vaccine reminder in patients aged under 65 who have a chronic condition (as described in Supplementary Table 1). We will randomise whole primary care practices to either a text messaging campaign to

For peer review only - http://bmjopen.bmj.com/site/about/guidelines.xhtml

eligible patients, or to standard care (chosen as the comparator group because practices currently employ a variety of methods to encourage at-risk patients for influenza vaccine).[8]

Aims and objectives

The aims of the study are (1) to develop the methodology for conducting cluster randomised trials of text messaging interventions using routine electronic health records; and (2) to implement a cluster randomised trial to test the effectiveness of using a text messaging influenza vaccine reminder in achieving an increase in influenza vaccine uptake in patients aged 18 to 65 with chronic condition compared to standard care.

Specific objectives are: (i) to evaluate the effect of text messaging influenza vaccination reminders in patients under 65 with chronic conditions; (ii) to evaluate the feasibility of recruiting and randomising practices to a text messaging intervention; (iii) to evaluate the feasibility of practice delivery of a text message intervention to eligible patients within a primary care setting, and (iv) to evaluate the feasibility of ascertaining practice data regarding text message delivery and flu vaccine uptake.

For peer review only

For peer review only - http://bmjopen.bmj.com/site/about/guidelines.xhtml

Methods

Study design

A cluster randomised trial[10 11] of a text messaging influenza vaccination reminder in primary care.

Study population

This study is taking place in English primary care, with general practices recruited from three settings: (i) the Clinical Practice Research Datalink (CPRD),[12] a primary care database based on Vision software and covering 8% of the UK population; (ii) ResearchOne, a primary care database based on TPP SystmOne software[13] and covering 7.6% of the UK population; (iii) iPLATO text messaging software users in London.[14]

Inclusion criteria

Eligible practices must currently use a text messaging system to communicate with patients about issues other than influenza vaccination (CPRD and London practices must use iPLATO,[14] ResearchOne practices must use TPP SystmOne text messaging software. Practices using other text messaging software will be excluded). Practices that used text messages for influenza vaccination reminders in the 2012/13 influenza season will be excluded.

Recruitment

Eligible practices in the CPRD (n=40) will be identified using the electronic health record and invited to the trial. All TPP SystmOne practices (n=2073) and London-based iPLATO practices (n=460) will be invited to the trial by the Primary Care Research Network. London iPLATO practices will also be contacted by iPLATO and TPP SystmOne practices will be targeted through SystmOne communications. The Primary Care Research Network[15] will be involved in practice recruitment to ensure that target sample size is reached.

For peer review only - http://bmjopen.bmj.com/site/about/guidelines.xhtml

Sample size calculation

The sample size calculation is based on a comparison between influenza vaccine uptake in the intervention group, in which a text message influenza vaccination reminder will be sent to eligible patients, and influenza vaccine uptake in the standard care group. A cluster-level analysis of the practice-specific proportions will be undertaken and the sample size calculation estimates the number of practices required for the study. A systematic review of reminders for influenza vaccination reported that reminders (of any type) for influenza vaccine among adults increased the uptake, with an odds ratio of 1.66, 95% confidence interval 1.31-2.09).[16] However, a text messaging vaccine reminder trial among children in the USA found only a 9% increase (RR=1.09, 95% confidence interval 1.04-1.15).[9] Therefore we have chosen to power our study for risk ratio of 1.075, representing an increase from 54% (uptake in the CPRD 2012/13) to 58%.

At 90% power and 5% significance, with an ICC by general practice of 0.024 and an average of 750 eligible patients per practice, we will require 100 practices (50 per arm) to identify a 7.5% increase in vaccine uptake from 54% to 58%. To account for differences in the number of eligible patients per practice and the proportion with mobile phone numbers recorded, we have chosen to recruit and randomise 150 practices to the study.

Assignment of interventions

Practices will be randomised on a 1:1 basis to the intervention and standard care groups. We will use block randomisation within each setting. In the CPRD and ResearchOne, we will stratify by region. The allocation sequence will be generated by an independent statistician who will be blinded to practice name and the allocation sequence will be

Box 1
"Hello *PATIENT NAME*, to reduce your risk of serious health problems from flu we recommend vaccination. Call *PRACTICE PHONE NUMBER* to book. *PRACTICE NAME"*

For peer review only - http://bmjopen.bmj.com/site/about/guidelines.xhtml

concealed from the study team. Once practices have been allocated, the trial coordinator will inform practices about their allocation and distribute study materials.

Blinding

Practices in this study will be randomised to intervention or standard care groups, but general practice staff will not be blinded. However, academic investigators and trial statisticians will manage and review data without knowledge of the allocation.

Interventions

Practices will be allocated to an intervention or standard care arm. Those in the intervention arm will be asked to send an influenza vaccination text message reminder to their under 65 at-risk patients. This includes patients who are under 65 with chronic heart disease, chronic neurological disease, diabetes, chronic kidney disease, chronic liver disease, chronic respiratory disease, immunosuppression, as set out by the Chief Medical Officer.[17] Practices will identify eligible patients based on their electronic medical records using their established systems. Practices use a standard set of Read codes (available from Primis[18]) to determine patient eligibility for influenza vaccination.

The message content that practices will be asked to send is shown in Box 1. This single text message was designed to be a pragmatic, uncomplicated intervention that would not discourage practices from participating, but that aims to address some of the key barriers to vaccination.[3] It was specifically designed to target more than one factor influencing behaviour: first, the text message represents a recommendation from the GP; second, it indicates the severity of flu and that the patient is susceptible to flu; third, it acts as a prompt to patients that might have simply forgotten to make an appointment.

Practices in the intervention group will receive guidance notes regarding delivery of the message (content, timing, eligible patients) and an incentive payment of £200. Practices will be instructed to continue with any other aspect of their seasonal flu campaign as planned, in

For peer review only - http://bmjopen.bmj.com/site/about/guidelines.xhtml

addition to the text message. An online questionnaire will be sent to practices in the intervention arm to ascertain feedback from practice staff about delivery of the intervention.

Practices allocated to the standard care group will be asked to continue with their seasonal flu campaign as planned.

Sub-study for patient feedback

A sub-study will be conducted in two participating intervention practices. Each practice will send an anonymous questionnaire to patients that were targeted with the text message reminder. This questionnaire will address patient acceptability of the text message.

Outcomes

The aims of the study are (1) to develop the methodology for conducting cluster randomised trials of text messaging interventions using routine electronic health records; and (2) to implement a cluster randomised trial to test the effectiveness of using a text messaging influenza vaccine reminder in achieving an increase in influenza vaccine uptake in patients aged 18 to 65 with chronic condition compared to standard care.

To address aim 2, we will examine the proportion of eligible patients that received the influenza vaccination by 31st December 2013 (primary outcome). This will be stratified by risk group, age and sex. A secondary outcome will be the proportion of eligible patients that were sent a text message to remind them about flu vaccine.

To address aim 1, we will measure the following as secondary outcomes:

- practice recruitment rate (based on number of practices invited across three trial settings, and the number recruited);
- the proportion of intervention practices and standard care practices that sent a text message to eligible patients;
- the proportion of eligible patients that were sent a message;
- the proportion of practices reporting any problems with delivering the message;

For peer review only - http://bmjopen.bmj.com/site/about/guidelines.xhtml

- the availability of data to examine text message receipt and vaccine uptake in each risk group, and
- the time and cost required to gather data.

In the patient sub-study, we will also examine the acceptability of text messaging to patients, by measuring the proportion of patients that reported problems with the text message.

Data collection methods

All patient-level outcomes will be evaluated through analysis of routinely collected data. In practices that contribute data to the CPRD or ResearchOne, relevant data will be extracted from the database using specified Read codes. Within London practices using iPLATO software, data will be extracted by the clinical software supplier of the practice, or the practices themselves.

We will extract data on age, sex, clinical risk group (based on historical data from the medical record and prescription data), vaccination reminder type (text, letter, phone call, face to face), vaccination uptake, death, transfer out of practice, flu and flu-like illness, and hospital admissions. Uptake and patient outcomes will be analysed from 1st September to 31st December 2013. All of these data items are routinely collected by general practices and stored in their electronic health record, and follow-up for practices will be complete using this method. Time and cost of data collection using these methods will be estimated. All data will be stripped of personal identifiers before being supplied to the study team, and will be stored securely as defined in the protocol.

We will record the number of practices approached, recruited and analysed. An online questionnaire, emailed to each practice in the intervention group, will ascertain practice acceptability of the intervention. The postal questionnaire to patients in the sub-study will estimate patient acceptability.

For peer review only - http://bmjopen.bmj.com/site/about/guidelines.xhtml
For peer review only

Statistical methods

A cluster-level analysis will be performed using practice specific proportions as observations. We will compare vaccine uptake in the intervention and standard care groups using a t test, with the size of clusters taken into account. A series of descriptive statistics will describe the methodological outcomes.

Sensitivity and subgroup analyses

Our primary analysis will be an intention to treat analysis. However, to account for any contamination between the standard care and intervention arms, we will carry out a per-protocol analysis.

Where available, we will also measure the difference in vaccine uptake, comparing practices that used the exact wording of the message in the study protocol with practices that used an alternative message. We hypothesise that practices using our message (based on behavioural theory) will have a higher uptake than an alternative designed by the practice. As this will be a non-randomised comparison, we will adjust for confounders.

Finally, we will compare the effectiveness of the text message based on the time of day that it was sent to patients. We hypothesise that messages sent in the late afternoon will have more effect that those sent at earlier times of day, when patients may not have the time to respond.

Hawthorne effect

The study design allows an evaluation of the generalisibility of the study population; practices that take part in the study can be compared to other practices outside of the trial that contribute data to ResearchOne and CPRD. We will test whether participation in the trial changes the behaviour of practices in their use of text messaging (Hawthorne effect).

This study will provide evidence regarding the effectiveness of text messaging reminders for influenza vaccine in patients under 65 with chronic conditions. The methodology here will be

For peer review only - http://bmjopen.bmj.com/site/about/guidelines.xhtml

applied to future cluster randomised trials of text messaging interventions within electronic heath records.

Ethics and dissemination

Ethical issues

Approvals

The study protocol (version 2.7, 11/02/2014) has received a favourable opinion from the National Research Ethics Service Committee – Surrey Borders (REC number 13/LO/0872) and has received assurance from participating Primary Care Trusts/ Clinical Commissioning Groups. All minor and major amendments to the protocol will be approved by the REC, and participating general practices will be notified of any protocol amendments relevant to them.

Informed consent

This is a cluster trial and informed consent will be obtained by the trial coordinator from GPs, who will act as the guardian of the cluster. A signed and dated informed consent form is required for participation in the trial. The risks and benefits of participation will be explained and GPs are free to decline to participate in the trial. Individual consent will not be sought. This is justified because participating practices already use text messaging to communicate with patients, though not systematically for influenza. Patients within these practices will have the opportunity to opt out of receiving messages if they do not wish to be contacted in this way.

Patient confidentiality

All patient-level data accessed in this study will be stripped of personal identifiers.

Access to data

The data will be accessed only by authorised persons from the London School of Hygiene and Tropical Medicine and research governance authorities to ensure that the study is being

For peer review only - http://bmjopen.bmj.com/site/about/guidelines.xhtml

For peer review only

carried out to acceptable standards. All will have a duty of confidentiality and no data will be disclosed outside the research site. All patient and practice-level information will be confidential.

Burden and risk to practices

Text messaging is widely used in everyday primary care and the only substantive change from non-study is the random allocation of a defined text message regarding influenza vaccine rather than the practice's own seasonal flu campaign. Patients can opt-out of receiving text messages from their practices, and as part of this study, we will monitor patient and practice complaints regarding the text message. Patients registered at practices in the intervention arm will receive one text message regarding their flu vaccine to minimise irritation to patients.

Participating practices must spend time identifying eligible patients and sending a text message. We are reducing this burden by (i) asking practices to use established procedures and software to identify eligible patients for vaccination. This is a task that they would perform annually in non-study conditions; (ii), we are including only practices that are familiar with the text messaging software, which minimises time spent in familiarisation with the software and sending messages; (iii) additional support is available to any practice through a guidance document and/or dedicated support helpline; (iv) we are providing an incentive payment (£200) to practices in the intervention group to encourage participation.

Practices may experience increased demand for influenza vaccination appointments or run out of vaccine supplies sooner than expected. The increase in uptake is unlikely to be large and will help the practice to achieve their Quality and Outcomes Framework target for vaccination, for which they receive payment.

Benefits for practices

For peer review only - http://bmjopen.bmj.com/site/about/guidelines.xhtml

Practices in the intervention arm will receive a £200 incentive to participate. If the text message is effective, then increased uptake will result in additional incentive payment to GPs by the government and will help them reach their Quality and Outcomes Framework targets. The results of this trial will allow practices to make an informed decision about spending of their budget on the seasonal influenza campaign.

Dissemination plans

The results of this trial will be published according to the guidelines of the CONSORT statement. The results will be published in a peer reviewed medical journal and presented at national conferences. The results will also be distributed to the Primary Care Research Network and to all participating general practices. Authorship for all publications will be based on the criteria defined by the International Committee of Medical Journal Editors.[19]

For peer review only

For peer review only - http://bmjopen.bmj.com/site/about/guidelines.xhtml

References

1. The flu immunisation programme 2013/14 (https://www.gov.uk/government/uploads/system/uploads/attachment_data/file/207008/130613_Flu_Letter_v_29_Gateway_GW_signed.pdf) accessed 25 March 2014.
2. Begum F, Pebody R, Department of Health. Seasonal influenza vaccine uptake amongst GP patient groups in England: Winter season 2011/12, 2012.
3. Wheelock A, Thomson A, Sevdalis N. Social and psychological factors underlying adult vaccination behavior: lessons from seasonal influenza vaccination in the US and the UK. *Expert review of vaccines* 2013;12(8):893-901.
4. mruk research Ltd. Flu & pneumococcal wave 4 - 2005/2006 post-campaign evaluation. Scottish Executive Social Research., 2006.
5. Keenan H, Campbell J, Evans PH. Influenza vaccination in patients with asthma: why is the uptake so low? *The British journal of general practice : the journal of the Royal College of General Practitioners* 2007;57(538):359-63.
6. Jacobson Vann Julie C, Szilagyi P. Patient reminder and recall systems to improve immunization rates. *Cochrane Database of Systematic Reviews*: John Wiley & Sons, Ltd, 2005.
7. Szilagyi PG, Bordley C, Vann JC, et al. Effect of patient reminder/recall interventions on immunization rates: A review. *JAMA* 2000;284(14):1820-7.
8. Dexter LJ, Teare MD, Dexter M, et al. Strategies to increase influenza vaccination rates: outcomes of a nationwide cross-sectional survey of UK general practice. *BMJ Open* 2012;2(3).
9. Stockwell MS, Kharbanda EO, Martinez RA, et al. Effect of a text messaging intervention on influenza vaccination in an urban, low-income pediatric and adolescent population: a randomized controlled trial. *JAMA* 2012;307(16):1702-8.
10. Campbell MK, Piaggio G, Elbourne DR, et al. Consort 2010 statement: extension to cluster randomised trials. *BMJ* 2012;345:e5661.
11. Weijer C, Grimshaw JM, Eccles MP, et al. The Ottawa Statement on the Ethical Design and Conduct of Cluster Randomized Trials. *PLoS Med* 2012;9(11):e1001346.
12. The Clinical Practice Research Datalink (CPRD) (www.cprd.com) accessed 1 December 2013.
13. TPP SystmOne (www.tpp-uk.com) accessed 1 December 2013.
14. iPLATO (www.iplato.net) accessed 17 April 2012.
15. The Primary Care Research Network (http://www.crncc.nihr.ac.uk/about_us/pcrn) accessed 25 March 2014.
16. Jacobson Vann Julie C, Szilagyi P. Patient reminder and recall systems to improve immunization rates. *Cochrane Database of Systematic Reviews*: John Wiley & Sons, Ltd, 2009.
17. McBride M, McArdle C, Timoney M. From the Chief Medical Officer: The seasonal influenza vaccination programme 2013/14. Department of Health, Social Services and Public Safety., 2013.
18. Primis (http://www.nottingham.ac.uk/primis/index.aspx) accessed 24th March 2014.
19. International Committee of Medical Journal Editors. Uniform requirements for manuscripts submitted to biomedical journals: Writing and editing for biomedical publication. *Journal of pharmacology & pharmacotherapeutics* 2010;1(1):42-58.

For peer review only - http://bmjopen.bmj.com/site/about/guidelines.xhtml

Authors' contributions

All authors were involved in the study design and in writing the manuscript.

Funding statement

This work was supported by the Wellcome Trust grant number 098504/Z/12/Z. The funder had no role in the trial design and will have no role in the collection, management, analysis and interpretation of data.

Competing interests statement

The authors declare no competing interests.

Acknowledgements

This trial is sponsored by the London School of Hygiene and Tropical Medicine, Keppel Street, London WC1E 7HT. The sponsor had no role in the study design and will have no role in data collection, management, analysis or interpretation of data. We would like to thank the LSHTM Clinical Trials Unit, Tim Collier, Noclor, Public Health England, the Cabinet Office, the Primary Care Research Network, the Department of Health, the Clinical Practice Research Datalink, ResearchOne, and iPLATO for their guidance and support.

For peer review only

For peer review only - http://bmjopen.bmj.com/site/about/guidelines.xhtml

Text messaging reminders for influenza vaccine in primary care: protocol for a cluster randomised controlled trial (TXT4FLUJAB)

Dr Emily Herrett (corresponding author, emily.herrett@lshtm.ac.uk)[1], Professor Tjeerd van Staa (tjeerd.vanstaa@lshtm.ac.uk)[1,2], Dr Caroline Free (caroline.free@lshtm.ac.uk)[1], Professor Liam Smeeth (liam.smeeth@lshtm.ac.uk)[1]

[1] London School of Hygiene and Tropical Medicine, Keppel Street, London, WC1E 7HT, UK

[2] Division of Pharmacoepidemiology and Clinical Pharmacology, Department of Pharmaceutical Sciences, Faculty of Sciences, Utrecht Institute for Pharmaceutical Sciences, Utrecht University, Utrecht, Netherlands

For peer review only - http://bmjopen.bmj.com/site/about/guidelines.xhtml

Abstract

Introduction: The UK government recommends that at least 75% of people aged under 65 with certain conditions receive an annual influenza vaccination. Primary care practices often fa~~i~~il short of this target and strategies to increase vaccine uptake are required. Text messaging reminders are already used in 30% of practices to remind patients about vaccination but there has been no trial addressing their effectiveness in increasing influenza vaccine uptake in the UK.

The aims of the study are (i) to develop the methodology for conducting cluster randomised trials of text messaging interventions utilising routine electronic health records~~to implement a cluster randomised trial to testassess the effectiveness of using a text messaging influenza vaccine reminder in achieving an increase in influenza vaccine uptake in patients aged 18 to 65 with chronic condition compared to standard care~~; and (ii) ~~-~~to assess the effectiveness of using a text messaging influenza vaccine reminder in achieving an increase in influenza vaccine uptake in patients aged 18 to 65 with chronic conditions, compared to standard care~~to develop the methodology for conducting cluster randomised trials of text messaging interventions utilising routine electronic health records~~.

Methods and analysis: This cluster randomised trial will recruit general practices across three settings in English primary care (Clinical Practice Research Datalink, ResearchOne and London iPLATO text messaging software users) and randomise them to either standard care or a text messaging campaign to eligible patients. Flu vaccine uptake will be ascertained using routinely collected, anonymised electronic patient records. This protocol outlines the proposed study design and analysis methods.

Ethics and dissemination: Results of this study will determine the effectiveness of text messaging vaccine reminders in primary care in increasing influenza vaccine uptake, and will strengthen the methodology for using electronic health records in cluster randomised trials of text messaging interventions. This trial was approved by Surrey Borders Ethics

For peer review only

Committee (13/LO/0872). The trial results will be disseminated at national conferences and published in a peer reviewed medical journal. The results will also be distributed to the Primary Care Research Network and to all participating general practices.

Trial registration: This study is registered at controlled-trials.com ISRCTN48840025, July 2013.

Strengths and limitations of this study

Strengths

- This trial is the first to evaluate the effectiveness of text messaging for influenza vaccine reminders in English primary care.
- Text messaging is cheap, quick, and has shown to be effective for appointment reminders.
- The study requires minimal input from practices as it utilises routinely collected electronic health records to gather vaccine uptake data.

Limitations

- There may be some contamination if general practices in the standard care arm choose to send a text message.

For peer review only

For peer review only - http://bmjopen.bmj.com/site/about/guidelines.xhtml

Introduction

Influenza morbidity and mortality causes a substantial financial burden to the NHS and to the UK as a whole. Influenza vaccine is safe and effective but is required annually because the circulating strain of virus changes each year. In the UK in 2012, the Chief Medical Officer (CMO) has recommended that at least 75% of elderly people (aged 65+) and 75% people under 65 with certain chronic conditions (e.g. chronic heart disease, diabetes, asthma, etc) should be vaccinated.[1]

While many primary care practices are achieving these targets for elderly patients (74% vaccinated in 2011/12), those set for patients with chronic conditions are not being met and have shown no substantial increases in the past decade;[2] and vaccine uptake in 2011/12 was 51.6% across the risk groups, (see Supplementary Table 1). Barriers to influenza vaccination in the UK include the lack of recommendation for vaccination by a healthcare worker,[3] lack of awareness of eligibility for vaccination,[4] low perceived susceptibility to and severity of influenza,[3-5] beliefs that the effectiveness of the vaccine is low, and beliefs about its side effects, safety, and pain.[3 5] Therefore sStrategies to increase flu vaccine uptake in these patients are required.

Several randomised trials have demonstrated the effectiveness of flu vaccine reminders delivered to patients by letter, postcard or telephone.[6 7] However, use of text messaging in the NHS for appointment reminders is increasing as it is cheap, quick and effective. Text messaging is already used in roughly 30% of practices to remind patients about their flu vaccine[8] but there has been no trial addressing its effectiveness in this context. Trials of text messaging in the US have shown some success as vaccine reminders,[9] but whether there is an effect in UK primary care is unknown.

Therefore, we are performing a randomised trial of a text messaging flu vaccine reminder in patients aged under 65 who have a chronic condition (as described in Supplementary Table 1). We will randomise whole primary care practices to either standard care or a text

For peer review only - http://bmjopen.bmj.com/site/about/guidelines.xhtml

messaging campaign to eligible patients, or to standard care (chosen - as the comparator group because practices currently employ a variety of methods to encourage at-risk patients for influenza vaccine).[8]

Aims and objectives

The aims of the study are (1) to develop the methodology for conducting cluster randomised trials of text messaging interventions using routine electronic health recordsto implement a cluster randomised trial to test the effectiveness of using a text messaging influenza vaccine reminder in achieving an increase in influenza vaccine uptake in patients aged 18 to 65 with chronic condition compared to standard care; and (2) to implement a cluster randomised trial to test the effectiveness of using a text messaging influenza vaccine reminder in achieving an increase in influenza vaccine uptake in patients aged 18 to 65 with chronic condition compared to standard care to develop the methodology for conducting cluster randomised trials of text messaging interventions using routine electronic health records.

Specific objectives are: (i) to evaluate the effect of text messaging influenza vaccination reminders in patients under 65 with chronic conditions; (ii) to evaluate the feasibility of recruiting and randomising practices to a text messaging intervention; (iii) to evaluate the feasibility of practice delivery of a text message intervention to eligible patients within a primary care setting, and (iv) to evaluate the feasibility of ascertaining practice data regarding text message delivery and flu vaccine uptake.

For peer review only - http://bmjopen.bmj.com/site/about/guidelines.xhtml

Methods

Study design

A cluster randomised trial[10 11] of a text messaging influenza vaccination reminder in primary care.

Study population

This study is taking place in English primary care, with general practices recruited from three settings: (i) the Clinical Practice Research Datalink (CPRD),[12] a primary care database based on Vision software and covering 8% of the UK population; (ii) ResearchOne, a primary care database based on TPP SystmOne software[13] and covering 7.6% of the UK population; (iii) iPLATO text messaging software users in London.[14]

Inclusion criteria

Eligible practices must currently use a text messaging system to communicate with patients about issues other than influenza vaccination (CPRD and London practices must use iPLATO,[14]; ResearchOne practices must use TPP SystmOne text messaging software. Practices using other text messaging software will be excluded). Practices that used text messages for influenza vaccination reminders in the 2012/13 influenza season will be excluded.

Recruitment

Eligible practices in the CPRD (n=40) will be identified using the electronic health record and invited to the trial. All TPP SystmOne practices (n=2073) and London-based iPLATO practices (n=460) will be invited to the trial by the Primary Care Research Network. London iPLATO practices will also be contacted by iPLATO and TPP SystmOne practices will be targeted through SystmOne communications. The Primary Care Research Network[15] will be involved in practice recruitment to ensure that target sample size is reached.

For peer review only - http://bmjopen.bmj.com/site/about/guidelines.xhtml

Sample size calculation

The sample size calculation is based on a comparison between influenza vaccine uptake in the intervention group, in which a text message influenza vaccination reminder will be sent to eligible patients, and influenza vaccine uptake in the standard care group. A cluster-level analysis of the practice-specific proportions will be undertaken and the sample size calculation estimates the number of practices required for the study. A systematic review of reminders for influenza vaccination reported that reminders (of any type) for influenza vaccine among adults increased the uptake, with an odds ratio of 1.66, 95% confidence interval 1.31-2.09).[16] However, a text messaging vaccine reminder trial among children in the USA found only a 9% increase (RR=1.09, 95% confidence interval 1.04-1.15).[9] Therefore we have chosen to power our study for risk ratio of 1.075, representing an increase from 54% (uptake in the CPRD 2012/13) to 58%.

At 90% power and 5% significance, with an ICC by general practice of 0.024 and an average of 750 eligible patients per practice, we will require 100 practices (50 per arm) to identify a 7.5% increase in vaccine uptake from 54% to 58%. To account for differences in the number of eligible patients per practice and ~~in~~ the proportion with mobile phone numbers recorded, we have chosen to recruit and randomise 150 practices to the study.

Assignment of interventions

Practices will be randomised on a 1:1 basis to the intervention and standard care groups. We will use block randomisation within each setting. In the CPRD and ResearchOne, we will stratify by region. The allocation sequence will be generated by an independent statistician who will be blinded to practice name and the allocation sequence will be

Box 1
"Hello *PATIENT NAME*, to reduce your risk of serious health problems from flu we recommend vaccination. Call *PRACTICE PHONE NUMBER* to book. *PRACTICE NAME"*

For peer review only - http://bmjopen.bmj.com/site/about/guidelines.xhtml

concealed from the study team. Once practices have been allocated, the trial coordinator will inform practices about their allocation and distribute study materials.

Blinding

Practices in this study will be randomised to intervention or standard care groups, but general practice staff will not be blinded. However, academic investigators and trial statisticians will manage and review data without knowledge of the allocation.

Interventions

Practices will be allocated to an intervention or standard care arm. Those in the intervention arm will be asked to send an influenza vaccination text message reminder to their under 65 at-risk patients. This includes patients who are under 65 with chronic heart disease, chronic neurological disease, diabetes, chronic kidney disease, chronic liver disease, chronic respiratory disease, immunosuppression, as set out by the Chief Medical Officer.[17] Practices will identify eligible patients based on their electronic medical records using their established systems. Practices use a standard set of Read codes (available from Primis[18]) to determine patient eligibility for influenza vaccination.

The message content that practices will be asked to send is shown in Box 1. This single text message was designed to be a pragmatic, uncomplicated intervention that would not discourage practices from participating, but that aims to address some of the key barriers to vaccination.[3] It was specifically designed to target more than one factor influencing behaviour: first, the text message represents a recommendation from the GP; second, it indicates the severity of flu and that the patient is susceptible to flu; third, it acts as a prompt to patients that might have simply forgotten to make an appointment.

Practices in the intervention group will receive guidance notes regarding delivery of the message (content, timing, eligible patients) and an incentive payment of £200. Practices will be instructed to continue with any other aspect of their seasonal flu campaign as planned, in

For peer review only - http://bmjopen.bmj.com/site/about/guidelines.xhtml

addition to the text message. An online questionnaire will be sent to practices in the intervention arm to ascertain feedback from practice staff about delivery of the intervention.

Practices allocated to the standard care group will be asked to continue with their seasonal flu campaign as planned.

Sub-study for patient feedback

A sub-study will be conducted in two participating intervention practices. Each practice will send an anonymous questionnaire to patients that were targeted with the text message reminder. This questionnaire will address patient acceptability of the text message.

Outcomes

The aims of the study are (1) to develop the methodology for conducting cluster randomised trials of text messaging interventions using routine electronic health records to implement a cluster randomised trial to test the effectiveness of using a text messaging influenza vaccine reminder in achieving an increase in influenza vaccine uptake in patients aged 18 to 65 with chronic condition compared to standard care; and (2) to develop the methodology for conducting cluster randomised trials of text messaging interventions using routine electronic health recordsto implement a cluster randomised trial to test the effectiveness of using a text messaging influenza vaccine reminder in achieving an increase in influenza vaccine uptake in patients aged 18 to 65 with chronic condition compared to standard care.

To address aim 12, we will examine the proportion of eligible patients that were sent a text message to remind them about flu vaccine, and the proportion that received the influenza vaccination by 31st December 2013 (primary outcome). This will be stratified by risk group, age and sex. A secondary outcome will be the proportion of eligible patients that were sent a text message to remind them about flu vaccine.

To address aim 21, we will measure the following as secondary outcomes:

For peer review only - http://bmjopen.bmj.com/site/about/guidelines.xhtml

- practice recruitment rate (based on number of practices invited across three trial settings, and the number recruited);
- the proportion of intervention practices and standard care practices that sent a text message to eligible patients;
- the proportion of eligible patients that were sent a message;
- the proportion of practices reporting any problems with delivering the message~~,~~;
- the availability of data to examine text message receipt and vaccine uptake in each risk group, and
- the time and cost required to gather data~~,~~.

In the patient sub-study, we will also examine the acceptability of text messaging to patients, by measuring the proportion of patients that reported problems with the text message.

Data collection methods

All patient-level outcomes will be evaluated through analysis of routinely collected data. In practices that contribute data to the CPRD or ResearchOne, relevant data will be extracted from the database using specified Read codes. Within London practices using iPLATO software, data will be extracted by ~~iPLATO using secure servers~~the clinical software supplier of the practice, or the practices themselves.

We will extract data on age, sex, clinical risk group (based on historical data from the medical record and prescription data), vaccination reminder type (text, letter, phone call, face to face), vaccination uptake, death, transfer out of practice, flu and flu-like illness, and hospital admissions. Uptake and patient outcomes will be analysed from 1st September to 31st December 2013. All of these data items are routinely collected by general practices and stored in their electronic health record, and follow-up for practices will be complete using this method. Time and cost of data collection using these methods will be estimated. All data will be stripped of personal identifiers before being supplied to the study team, and will be stored securely as defined in the protocol.

For peer review only

We will record the number of practices approached, recruited and analysed. An online questionnaire, emailed to each practice in the intervention group, will ascertain practice acceptability of the intervention. The postal questionnaire to patients in the sub-study will estimate patient acceptability.

Statistical methods

A cluster-level analysis will be performed using practice specific proportions as observations. We will compare vaccine uptake in the intervention and standard care groups using a t test, with the size of clusters taken into account. A series of descriptive statistics will describe the methodological outcomes.

Sensitivity and subgroup analyses

Our primary analysis will be an intention to treat analysis. However, to account for any contamination between the standard care and intervention arms, we will carry out a per-protocol analysis.

Where available, we will also measure the difference in vaccine uptake, comparing practices that used the exact wording of the message in the study protocol with practices that used an alternative message. We hypothesise that practices using our message (based on behavioural theory) will have a higher uptake than an alternative designed by the practice. As this will be a non-randomised comparison, we will adjust for confounders.

Finally, we will compare the effectiveness of the text message based on the time of day that it was sent to patients. We hypothesise that messages sent in the late afternoon will have more effect that those sent at earlier times of day, when patients may not have the time to respond.

For peer review only - http://bmjopen.bmj.com/site/about/guidelines.xhtml

Hawthorne effect

The study design allows an evaluation of the generalisibility of the study population; practices that take part in the study can be compared to other practices outside of the trial that contribute data to ResearchOne and CPRD. We will test whether participation in the trial changes the behaviour of practices in their use of text messaging (Hawthorne effect).

This study will provide evidence regarding the effectiveness of text messaging reminders for influenza vaccine in patients under 65 with chronic conditions. The methodology here will be applied to future cluster randomised trials of text messaging interventions within electronic heath records.

Ethics and dissemination

Ethical issues

Approvals

The study protocol (version 2.7, 11/02/2014) has received a favourable opinion from the National Research Ethics Service Committee – Surrey Borders (REC number 13/LO/0872) and has received assurance from participating Primary Care Trusts/ Clinical Commissioning Groups. All minor and major amendments to the protocol will be approved by the REC, and participating general practices will be notified of any protocol amendments relevant to them.

Informed consent

This is a cluster trial and informed consent will be obtained by the trial coordinator from GPs, who will act as the guardian of the cluster. A signed and dated informed consent form is required for participation in the trial. The risks and benefits of participation will be explained and GPs are free to decline to participate in the trial. Individual consent will not be sought. This is justified because participating practices already use text messaging to communicate with patients, though not systematically for influenza. Patients within these practices will

For peer review only - http://bmjopen.bmj.com/site/about/guidelines.xhtml

have the opportunity to opt out of receiving messages if they do not wish to be contacted in this way.

Patient confidentiality

All patient-level data accessed in this study will be stripped of personal identifiers.

Access to data

The data will be accessed only by authorised persons from the London School of Hygiene and Tropical Medicine and research governance authorities to ensure that the study is being carried out to acceptable standards. All will have a duty of confidentiality and no data will be disclosed outside the research site. All patient and practice-level information will be confidential.

Burden and risk to practices

Text messaging is widely used in everyday primary care and the only substantive change from non-study is the random allocation of a defined text message regarding influenza vaccine rather than the practice's own seasonal flu campaign. Patients can opt-out of receiving text messages from their practices, and as part of this study, we will monitor patient and practice complaints regarding the text message. Patients registered at practices in the intervention arm will receive one text message regarding their flu vaccine to minimise irritation to patients.

Participating practices must spend time identifying eligible patients and sending a text message. We are reducing this burden by (i) asking practices to use established procedures and software to identify eligible patients for vaccination. This is a task that they would perform annually in non-study conditions; (ii), we are including only practices that are familiar with the text messaging software, which minimises time spent in familiarisation with the software and sending messages; (iii) additional support is available to any practice through a guidance document and/or dedicated support helpline; (iv) we are providing an incentive payment (£200) to practices in the intervention group to encourage participation.

For peer review only - http://bmjopen.bmj.com/site/about/guidelines.xhtml

Practices may experience increased demand for influenza vaccination appointments or run out of vaccine supplies sooner than expected. The increase in uptake is unlikely to be large and will help the practice to achieve their Quality and Outcomes Framework target for vaccination, for which they receive payment.

Benefits for practices

Practices in the intervention arm will receive a £200 incentive to participate. If the text message is effective, then increased uptake will result in additional incentive payment to GPs by the government and will help them reach their Quality and Outcomes Framework targets. The results of this trial will allow practices to make an informed decision about spending of their budget on the seasonal influenza campaign.

Dissemination plans

The results of this trial will be published according to the guidelines of the CONSORT statement. The results will be published in a peer reviewed medical journal and presented at national conferences. The results will also be distributed to the Primary Care Research Network and to all participating general practices. Authorship for all publications will be based on the criteria defined by the International Committee of Medical Journal Editors.[19]

For peer review only - http://bmjopen.bmj.com/site/about/guidelines.xhtml

References

1. The flu immunisation programme 2013/14 (https://www.gov.uk/government/uploads/system/uploads/attachment_data/file/207008/130613_Flu_Letter_v_29_Gateway_GW_signed.pdf) accessed 25 March 2014.
2. Begum F, Pebody R, Department of Health. Seasonal influenza vaccine uptake amongst GP patient groups in England: Winter season 2011/12, 2012.
3. Wheelock A, Thomson A, Sevdalis N. Social and psychological factors underlying adult vaccination behavior: lessons from seasonal influenza vaccination in the US and the UK. *Expert review of vaccines* 2013;12(8):893-901.
4. mruk research Ltd. Flu & pneumococcal wave 4 - 2005/2006 post-campaign evaluation. Scottish Executive Social Research., 2006.
5. Keenan H, Campbell J, Evans PH. Influenza vaccination in patients with asthma: why is the uptake so low? *The British journal of general practice : the journal of the Royal College of General Practitioners* 2007;57(538):359-63.
6. Jacobson Vann Julie C, Szilagyi P. Patient reminder and recall systems to improve immunization rates. *Cochrane Database of Systematic Reviews*: John Wiley & Sons, Ltd, 2005.
7. Szilagyi PG, Bordley C, Vann JC, Chelminski A, Kraus RM, Margolis PA, et al. Effect of patient reminder/recall interventions on immunization rates: A review. *JAMA* 2000;284(14):1820-7.
8. Dexter LJ, Teare MD, Dexter M, Siriwardena AN, Read RC. Strategies to increase influenza vaccination rates: outcomes of a nationwide cross-sectional survey of UK general practice. *BMJ Open* 2012;2(3).
9. Stockwell MS, Kharbanda EO, Martinez RA, Vargas CY, Vawdrey DK, Camargo S. Effect of a text messaging intervention on influenza vaccination in an urban, low-income pediatric and adolescent population: a randomized controlled trial. *JAMA* 2012;307(16):1702-8.
10. Campbell MK, Piaggio G, Elbourne DR, Altman DG. Consort 2010 statement: extension to cluster randomised trials. *BMJ* 2012;345:e5661.
11. Weijer C, Grimshaw JM, Eccles MP, McRae AD, White A, Brehaut JC, et al. The Ottawa Statement on the Ethical Design and Conduct of Cluster Randomized Trials. *PLoS Med* 2012;9(11):e1001346.
12. The Clinical Practice Research Datalink (CPRD) (www.cprd.com) accessed 1 December 2013.
13. TPP SystmOne (www.tpp-uk.com) accessed 1 December 2013.
14. iPLATO (www.iplato.net) accessed 17 April 2012.
15. The Primary Care Research Network (http://www.crncc.nihr.ac.uk/about_us/pcrn) accessed 25 March 2014.
16. Jacobson Vann Julie C, Szilagyi P. Patient reminder and recall systems to improve immunization rates. *Cochrane Database of Systematic Reviews*: John Wiley & Sons, Ltd, 2009.
17. McBride M, McArdle C, Timoney M. From the Chief Medical Officer: The seasonal influenza vaccination programme 2013/14. Department of Health, Social Services and Public Safety., 2013.
18. Primis (http://www.nottingham.ac.uk/primis/index.aspx) accessed 24th March 2014.
19. International Committee of Medical Journal Editors. Uniform requirements for manuscripts submitted to biomedical journals: Writing and editing for biomedical publication. *Journal of pharmacology & pharmacotherapeutics* 2010;1(1):42-58.

For peer review only - http://bmjopen.bmj.com/site/about/guidelines.xhtml

Authors' contributions

All authors were involved in the study design and in writing the manuscript.

For peer review only

Funding statement

This work was supported by the Wellcome Trust grant number 098504/Z/12/Z. The funder had no role in the trial design and will have no role in the collection, management, analysis and interpretation of data.

Competing interests statement

The authors declare no competing interests.

Acknowledgements

This trial is sponsored by the London School of Hygiene and Tropical Medicine, Keppel Street, London WC1E 7HT. The sponsor had no role in the study design and will have no role in data collection, management, analysis or interpretation of data. We would like to thank the LSHTM Clinical Trials Unit, Tim Collier, Noclor, Public Health England, the Cabinet Office, the Primary Care Research Network, the Department of Health, the Clinical Practice Research Datalink, Resear~~a~~chOne, and iPLATO for their guidance and support.

SPIRIT 2013 Checklist: Recommended items to address in a clinical trial protocol and related documents*

Section/item	Item No	Description	Addressed on page number
**Administrative information**			
Title	1	Descriptive title identifying the study design, population, interventions, and, if applicable, trial acronym	___1________
Trial registration	2a	Trial identifier and registry name. If not yet registered, name of intended registry	___4________
	2b	All items from the World Health Organization Trial Registration Data Set	___1-16_____
Protocol version	3	Date and version identifier	___12_______
Funding	4	Sources and types of financial, material, and other support	___16_______
Roles and responsibilities	5a	Names, affiliations, and roles of protocol contributors	___1, 16_____
	5b	Name and contact information for the trial sponsor	___16_______
	5c	Role of study sponsor and funders, if any, in study design; collection, management, analysis, and interpretation of data; writing of the report; and the decision to submit the report for publication, including whether they will have ultimate authority over any of these activities	___16_______
	5d	Composition, roles, and responsibilities of the coordinating centre, steering committee, endpoint adjudication committee, data management team, and other individuals or groups overseeing the trial, if applicable (see Item 21a for data monitoring committee)	___N/A______

For peer review only - http://bmjopen.bmj.com/site/about/guidelines.xhtml

For peer review only

**Introduction**			
Background and rationale	6a	Description of research question and justification for undertaking the trial, including summary of relevant studies (published and unpublished) examining benefits and harms for each intervention	___4_________
	6b	Explanation for choice of comparators	___4/5_______
Objectives	7	Specific objectives or hypotheses	___5_________
Trial design	8	Description of trial design including type of trial (eg, parallel group, crossover, factorial, single group), allocation ratio, and framework (eg, superiority, equivalence, noninferiority, exploratory)	___6,7_______
**Methods: Participants, interventions, and outcomes**			
Study setting	9	Description of study settings (eg, community clinic, academic hospital) and list of countries where data will be collected. Reference to where list of study sites can be obtained	___6_________
Eligibility criteria	10	Inclusion and exclusion criteria for participants. If applicable, eligibility criteria for study centres and individuals who will perform the interventions (eg, surgeons, psychotherapists)	___6_________
Interventions	11a	Interventions for each group with sufficient detail to allow replication, including how and when they will be administered	___8_________
	11b	Criteria for discontinuing or modifying allocated interventions for a given trial participant (eg, drug dose change in response to harms, participant request, or improving/worsening disease)	___N/A______
	11c	Strategies to improve adherence to intervention protocols, and any procedures for monitoring adherence (eg, drug tablet return, laboratory tests)	___N/A______
	11d	Relevant concomitant care and interventions that are permitted or prohibited during the trial	____8________
Outcomes	12	Primary, secondary, and other outcomes, including the specific measurement variable (eg, systolic blood pressure), analysis metric (eg, change from baseline, final value, time to event), method of aggregation (eg, median, proportion), and time point for each outcome. Explanation of the clinical relevance of chosen efficacy and harm outcomes is strongly recommended	____9________
Participant timeline	13	Time schedule of enrolment, interventions (including any run-ins and washouts), assessments, and visits for participants. A schematic diagram is highly recommended (see Figure)	Supplementary material

For peer review only - http://bmjopen.bmj.com/site/about/guidelines.xhtml

Sample size	14	Estimated number of participants needed to achieve study objectives and how it was determined, including clinical and statistical assumptions supporting any sample size calculations	___7_________
Recruitment	15	Strategies for achieving adequate participant enrolment to reach target sample size	___6________
**Methods: Assignment of interventions (for controlled trials)**			
Allocation:			
Sequence generation	16a	Method of generating the allocation sequence (eg, computer-generated random numbers), and list of any factors for stratification. To reduce predictability of a random sequence, details of any planned restriction (eg, blocking) should be provided in a separate document that is unavailable to those who enrol participants or assign interventions	___7/8_________
Allocation concealment mechanism	16b	Mechanism of implementing the allocation sequence (eg, central telephone; sequentially numbered, opaque, sealed envelopes), describing any steps to conceal the sequence until interventions are assigned	___7/8_______
Implementation	16c	Who will generate the allocation sequence, who will enrol participants, and who will assign participants to interventions	___7/8______
Blinding (masking)	17a	Who will be blinded after assignment to interventions (eg, trial participants, care providers, outcome assessors, data analysts), and how	___7/8______
	17b	If blinded, circumstances under which unblinding is permissible, and procedure for revealing a participant's allocated intervention during the trial	___N/A________
**Methods: Data collection, management, and analysis**			
Data collection methods	18a	Plans for assessment and collection of outcome, baseline, and other trial data, including any related processes to promote data quality (eg, duplicate measurements, training of assessors) and a description of study instruments (eg, questionnaires, laboratory tests) along with their reliability and validity, if known. Reference to where data collection forms can be found, if not in the protocol	____10_______
	18b	Plans to promote participant retention and complete follow-up, including list of any outcome data to be collected for participants who discontinue or deviate from intervention protocols	____10_______

For peer review only - http://bmjopen.bmj.com/site/about/guidelines.xhtml

Data management	19	Plans for data entry, coding, security, and storage, including any related processes to promote data quality (eg, double data entry; range checks for data values). Reference to where details of data management procedures can be found, if not in the protocol	__10_________
Statistical methods	20a	Statistical methods for analysing primary and secondary outcomes. Reference to where other details of the statistical analysis plan can be found, if not in the protocol	__11_________
	20b	Methods for any additional analyses (eg, subgroup and adjusted analyses)	__11_________
	20c	Definition of analysis population relating to protocol non-adherence (eg, as randomised analysis), and any statistical methods to handle missing data (eg, multiple imputation)	__11________
**Methods: Monitoring**			
Data monitoring	21a	Composition of data monitoring committee (DMC); summary of its role and reporting structure; statement of whether it is independent from the sponsor and competing interests; and reference to where further details about its charter can be found, if not in the protocol. Alternatively, an explanation of why a DMC is not needed	__ N/A ________
	21b	Description of any interim analyses and stopping guidelines, including who will have access to these interim results and make the final decision to terminate the trial	___N/A________
Harms	22	Plans for collecting, assessing, reporting, and managing solicited and spontaneously reported adverse events and other unintended effects of trial interventions or trial conduct	___10, 13____
Auditing	23	Frequency and procedures for auditing trial conduct, if any, and whether the process will be independent from investigators and the sponsor	___ N/A _______
**Ethics and dissemination**			
Research ethics approval	24	Plans for seeking research ethics committee/institutional review board (REC/IRB) approval	___12________
Protocol amendments	25	Plans for communicating important protocol modifications (eg, changes to eligibility criteria, outcomes, analyses) to relevant parties (eg, investigators, REC/IRBs, trial participants, trial registries, journals, regulators)	___12_________

For peer review only - http://bmjopen.bmj.com/site/about/guidelines.xhtml

Consent or assent	26a	Who will obtain informed consent or assent from potential trial participants or authorised surrogates, and how (see Item 32)	___12_________
	26b	Additional consent provisions for collection and use of participant data and biological specimens in ancillary studies, if applicable	___N/A_______
Confidentiality	27	How personal information about potential and enrolled participants will be collected, shared, and maintained in order to protect confidentiality before, during, and after the trial	____13_______
Declaration of interests	28	Financial and other competing interests for principal investigators for the overall trial and each study site	____16_______
Access to data	29	Statement of who will have access to the final trial dataset, and disclosure of contractual agreements that limit such access for investigators	____13______
Ancillary and post-trial care	30	Provisions, if any, for ancillary and post-trial care, and for compensation to those who suffer harm from trial participation	___N/A________
Dissemination policy	31a	Plans for investigators and sponsor to communicate trial results to participants, healthcare professionals, the public, and other relevant groups (eg, via publication, reporting in results databases, or other data sharing arrangements), including any publication restrictions	___14________
	31b	Authorship eligibility guidelines and any intended use of professional writers	___14________
	31c	Plans, if any, for granting public access to the full protocol, participant-level dataset, and statistical code	___N/A_______
**Appendices**			
Informed consent materials	32	Model consent form and other related documentation given to participants and authorised surrogates	supplementary material
Biological specimens	33	Plans for collection, laboratory evaluation, and storage of biological specimens for genetic or molecular analysis in the current trial and for future use in ancillary studies, if applicable	__N/A________

*It is strongly recommended that this checklist be read in conjunction with the SPIRIT 2013 Explanation & Elaboration for important clarification on the items. Amendments to the protocol should be tracked and dated. The SPIRIT checklist is copyrighted by the SPIRIT Group under the Creative Commons "Attribution-NonCommercial-NoDerivs 3.0 Unported" license.

For peer review only - http://bmjopen.bmj.com/site/about/guidelines.xhtml

Supplementary Table 1. Risk groups for influenza vaccination

**Risk groups for influenza vaccination**
Chronic respiratory disease
Chronic heart disease
Chronic renal disease
Diabetes
Immunosuppression
Liver disease
Chronic neurological disease
Carer
Pregnancy*

*not included in the TXT4FLUJAB study for ethical reasons

For peer review only

Supplementary Table 2. Schedule of enrolment, interventions and assessments

	Study period				
	**Enrolment**	**Allocation**	**Post allocation**		**Closeout**
**Time point**	**Aug 2013**	**Sept 2013**	**Sep to Dec 2013**	**Dec 2013**	**May 2014**
**Enrolment:**					
Eligibility screen	x				
Informed consent	x				
Allocation		x			
**Interventions:**					
Intervention			x		
Control			x		
**Assessments:**					
Practices complete questionnaire				x	
Researchers extract relevant data from practice (age, sex, clinical risk group, influenza vaccination reminder types, vaccine uptake)					x
Questionnaire to patients in the substudy				x	

For peer review only

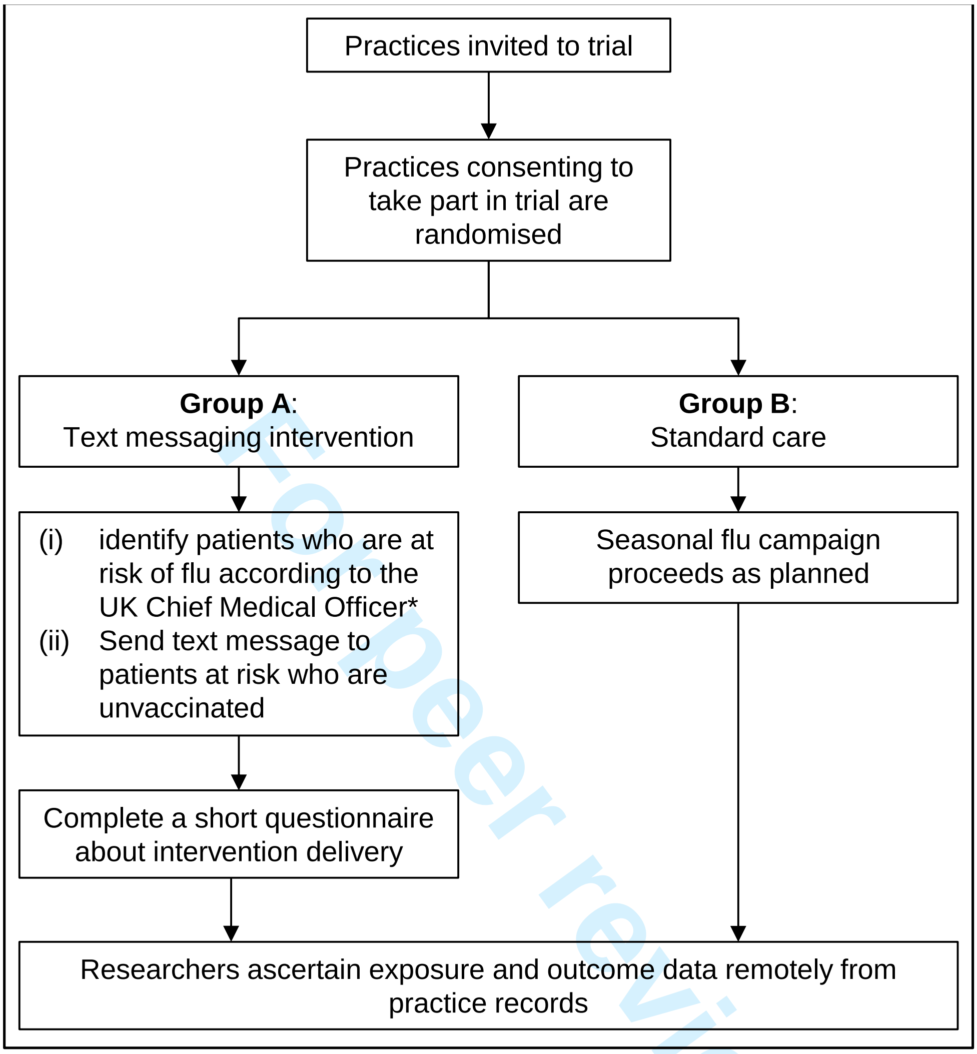

Supplementary Figure 1. Flowchart of TXT4FLUJAB study

For peer review only
